# Perspectives in Peptide-Based Vaccination Strategies for Syndrome Coronavirus 2 Pandemic

**DOI:** 10.3389/fphar.2020.578382

**Published:** 2020-12-03

**Authors:** Concetta Di Natale, Sara La Manna, Ilaria De Benedictis, Paola Brandi, Daniela Marasco

**Affiliations:** ^1^ Department of Pharmacy, University of Naples Federico II, Naples, Italy; ^2^ Center for Advanced Biomaterial for Health Care (CABHC), Istituto Italiano Di Tecnologia, Naples, Italy; ^3^ Interdisciplinary Research Centre on Biomaterials (CRIB) and Dipartimento di Ingegneria Chimica, Dei Materiali e Della Produzione Industriale, University of Naples Federico II, Naples, Italy; ^4^ Centro Nacional De Investigaciones Cardiovasculares Carlos III (CNIC), Madrid, Spain

**Keywords:** SARS-Cov-2, COVID-19, peptide-based vaccine, clinical trials, peptide on market

## Abstract

At the end of December 2019, an epidemic form of respiratory tract infection now named COVID-19 emerged in Wuhan, China. It is caused by a newly identified viral pathogen, the severe acute respiratory syndrome coronavirus (SARS-CoV-2), which can cause severe pneumonia and acute respiratory distress syndrome. On January 30, 2020, due to the rapid spread of infection, COVID-19 was declared as a global health emergency by the World Health Organization. Coronaviruses are enveloped RNA viruses belonging to the family of Coronaviridae, which are able to infect birds, humans and other mammals. The majority of human coronavirus infections are mild although already in 2003 and in 2012, the epidemics of SARS-CoV and Middle East Respiratory Syndrome coronavirus (MERS-CoV), respectively, were characterized by a high mortality rate. In this regard, many efforts have been made to develop therapeutic strategies against human CoV infections but, unfortunately, drug candidates have shown efficacy only into *in vitro* studies, limiting their use against COVID-19 infection. Actually, no treatment has been approved in humans against SARS-CoV-2, and therefore there is an urgent need of a suitable vaccine to tackle this health issue. However, the puzzled scenario of biological features of the virus and its interaction with human immune response, represent a challenge for vaccine development. As expected, in hundreds of research laboratories there is a running out of breath to explore different strategies to obtain a safe and quickly spreadable vaccine; and among others, the peptide-based approach represents a turning point as peptides have demonstrated unique features of selectivity and specificity toward specific targets. Peptide-based vaccines imply the identification of different epitopes both on human cells and virus capsid and the design of peptide/peptidomimetics able to counteract the primary host-pathogen interaction, in order to induce a specific host immune response. SARS-CoV-2 immunogenic regions are mainly distributed, as well as for other coronaviruses, across structural areas such as spike, envelope, membrane or nucleocapsid proteins. Herein, we aim to highlight the molecular basis of the infection and recent peptide-based vaccines strategies to fight the COVID-19 pandemic including their delivery systems.

## Introduction

Coronavirus disease (COVID-19) appeared for the first time in Wuhan, China: here different pneumonia cases of unknown etiology were identified and linked to the city’s seafood market (Zhu N. et al., 2020; Zhou et al., 2020). The alarmingly quick spread of the infection resulted in a worldwide health crisis that was declared a pandemic by the World Health Organization (WHO) on March 11, 2020. To date, more than 12 million cases have been identified in at least 190 different countries and unfortunately more than 636,000 people have died (Director, 2020). Considering that COVID-19 is a novel disease, several studies are currently elucidating details of its spreading. It is well known now that its diffusion is extremely rapid, even faster than influenza. Even if people are more contagious when they express symptoms, one of the most relevant issue of this disease is the so-called pre-symptomatic transmission; indeed, infected people can transmit the virus before symptoms appear and it has been recently reported that more than 40% of infected people show no symptoms.

Severe acute respiratory syndrome coronavirus 2 (SARS-CoV-2) has been identified as the causative agent of the COVID-19^4^. Human coronaviruses HCoV-229E and HCoVNL63 belong to the alpha family, while among beta coronaviruses MERS-CoV, SARS-CoV and SARS-CoV-2^5^ are encountered that resulted in lethal epidemics, with severe respiratory syndrome in humans (Wong et al., 2004; Yin and Wunderink, 2018; Director, 2020; Gorbalenya et al., 2020; Guan et al., 2020; Lauer et al., 2020; Xu et al., 2020). In particular, SARS-CoV-2 is transmitted primarily through respiratory droplets and the incubation time is about 5–6 days before symptoms onset, when SARS-CoV-2 viral load reaches its peak (Guan et al., 2020; Lauer et al., 2020). The pathological effects of COVID-19 are heterogeneous and the majority of infected people exhibit only moderate symptoms (Xu et al., 2020), but it can also progress to severe pneumonia, causing hypoxia and acute respiratory distress syndrome (ARDS), septic shock and/or multiple organ failure. Like SARS-CoV, SARS-CoV-2 infection leads to aggressive inflammatory responses causing a dysregulated host response that in turn is responsible for airway damage (Wong et al., 2004). Indeed, in response to viral and/or secondary infections, the immune system promotes an uncontrolled inflammation, resulting in a “cytokine storm” (Zhang B. et al., 2020). Furthermore, patients can develop both cardiovascular complications as heart failure, myocarditis or cardiac arrhythmias (Guo et al., 2020) and renal impairment (Chu et al., 2005).

Currently, there are no drugs approved for the treatment of this virus, but several old drugs are being used to counteract the symptoms of COVID-19 and to fight the virus, such as antiviral (lopinavir/ritonavir, remdesivir), anti-HIV (antiretroviral), antimalarial (chloroquine/hydroxychloroquine), antibiotic (azithromycin), corticosteroids and immunomodulatory agents (tocilizumab, adalimumab) (Ferner and Aronson, 2020; Popescu and Fischer, 2020; Wang M. et al., 2020).

Since COVID-19 is a very recent pandemic, large global efforts are being made to develop a vaccine as protection against COVID-19, although vaccine development timelines are difficult to be predicted and no one has completed clinical trials yet. The traditional vaccines are linked to several disadvantages; indeed, they can cause allergic and autoimmune reactions, have low stability and need storage at a cold temperature. To overcome these difficulties, a promising strategy may be the development of peptide-based vaccines. The use of peptides as antigens is a novel approach that involves minimal microbial components to stimulate adaptive immunity against a microorganism. These vaccines are able to target very specific epitopes removing the risks associated with allergic and autoimmune responses (Li et al., 2014). In this review we will describe SARS-CoV-2 immunogenic regions and then we will focus our attention on the recent advantages of peptide-based vaccines against COVID-19.

## COVID-19

### Host Infection and Inflammatory Immune Responses

For RNA viruses, as SARS-CoV-2, innate immune signaling is initiated through the engagement of pattern recognition receptors (PRRs) by viral RNA and in particular via cytosolic retinoic acid-inducible gene I (RIG-I)- like receptors (RLRs) and extracellular and endosomal toll-like receptors (TLRs) that initiate the cascade secretion of cytokines(Lester and Li, 2014; Kell and Gale, 2015). Subsequently, pro-inflammatory cytokines and chemokines as interleukin (IL)-6, interferon (IFN) and monocyte chemoattractant protein (MCP)-1, are released into the blood of patients (Guan et al., 2020) and are able to attract immune cells, in particular monocytes and T lymphocytes, from the blood toward the infected sites. Among dysregulated cytokines, IL-6 presents increasing levels over time, more elevated in non-survivors than in survivors (Zhou et al., 2020). The cytokine storm can contribute to vascular permeability and leakage, which participate in the pathophysiology of hypotension and pulmonary dysfunction in acute respiratory distress syndrome (ARDS). The key role of IL-6 in this cascade has prompted the evaluation of IL-6 antagonists such as several specific monoclonal antibodies as tocilizumab, sarilumab and siltuximab for the treatment of severe cases of COVID-19 (Moore and June, 2020). At the cellular level, patients present higher leukocyte and neutrophil counts, neutrophil/lymphocyte ratio (NLR), lower percentages of monocytes, eosinophils, basophils and lymphocytes (Qin et al., 2020) with respect to healthy subjects. In fact, both B- and T-cells play an important role in the control of the infection. In particular, CD4^+^ T-cells are needed to prime both CD8^+^ T- and B-cells. Specifically, CD8^+^ T-cells are responsible for the elimination of virus-infected cells (Diao et al., 2020), as demonstrated by mice depleted of CD4^+^ T-cells that exhibited a reduced clearance of the SARS-CoV (Channappanavar et al., 2014) as well as by specific CoV-memory T-cells found in people affected by SARS-CoV (Yang et al., 2006). Altogether, T-cells can be considered as crucial targets for the development of vaccines, as well as B-cells that are important in the response against coronaviruses, since the administration of serum samples from infected people containing antibodies against different viral proteins demonstrated being effective to ameliorate the clinical pictures of patients (Cheng et al., 2005) (see below).

### Structural Proteins, Homologies and Differences Between Human Coronaviruses

**FIGURE 1:**
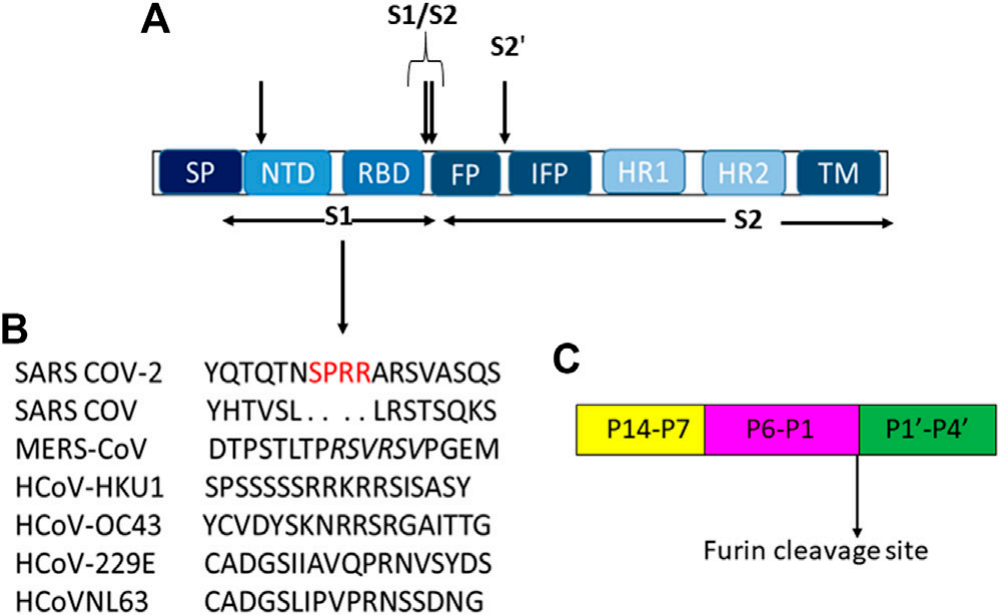
**(A)** Schematic representation of modular structure of SARS-CoV S-protein and its furin cleavage sites indicated by arrows. SP = signal peptide. **(B)** S1 cleavage furin site on human coronaviruses, the novel sites in SARS-CoV-2 is underlined in red while the furin cleavage on MERS is italicized. **(C)** Typical furin cleavage site.

All coronaviruses encode for four or five structural proteins known as spike (S), membrane (M), envelope (E), nucleocapsid (N) and hemagglutinin-esterase (HE) proteins (Liang et al., 2020). Viral structural proteins are components of the mature viruses, while non-structural proteins are encoded and expressed in infected cells but they are not assembled in the virion (Lazarowitz et al., 1971). S is an homotrimeric glycoprotein located on the viral envelope surface and hence crucial for the attachment to host cells (Li, 2016); during entry it involves two functional subunits: S1 to bind the host cell receptor and S2 to fuse with the host cellular membranes (Le Coupanec et al., 2015; Millet and Whittaker, 2014) (Figure 1). These subunits are generated by a host protease, which is generally a furin-like enzyme. Furin proteases are members of serine protein convertases (PCs). Usually, PCs cleave precursor proteins at specific single or paired basic amino acids within the motif (arginine/lysine(R/K)-(2X)n-(R/K))↓(Seidah and Chrétien, 1999; Maret et al., 2012), where n stands for spacer amino acids and ↓ to delineate the cleavage site. They are able to cleave specifically viral envelope glycoproteins, increasing cell membrane fusion (Gui et al., 2017; Pallesen et al., 2017; Song et al., 2018; Coutard et al., 2020). The canonical (R/K)-(2X)n-(R/K))↓ motifs for several human coronaviruses are reported in Table 1.

**TABLE 1:** Canonical alpha and beta HCoVs furin cleavage motif, ↓ indicates cut site of the protease.

Alpha HCoVs	Beta HCoVs	S1/S2, site 1	S1/S2, site 1	S2′
HCoV-229E	—	IAVQPR↓NVSYD	—	SRVAGR↓SA
HCoVNL63	—	IPVRPR↓NSSDN	—	SRIAGR↓SA
—	HCoV-HKU1	SRRKRR↓SISA	—	CGSSSR↓SF
—	HCoV-OC43	KNRRSR↓GAITT	—	SKASSR↓SA
—	MERS-CoV	TPRSCR↓SVPG	—	GSRSAR↓SA
—	SARS-CoV	TVSLLR↓STGQ	IAY↓TMS	LKPTKR↓SF
—	SARS-CoV-2	SPRRAR↓SVAS	IAY↓TMS	SKPSKR↓SF

For SARS-CoV-2, this cleavage activates the S protein for membrane fusion mechanism causing an irreversible conformational change (Belouzard et al., 2009; Park et al., 2016; Walls et al., 2016a). Spike of SARS-CoV-2 adopts an architecture similar to that of the SARS-CoV (Figure 1A), with S1 constituted by four sub-domains: NTD (N-terminal domain), receptor binding domain (RBD), and two CTDs (C-terminal domains). The S2-protein domain, instead, covers a second proteolytic site (S2′) upstream of the fusion peptide (FP), an internal fusion peptide (IFP), that is similar in SARS-CoV and SARS-CoV-2, and two heptad-repeat domains preceding the transmembrane domain (TM) (Wang D. et al., 2020).

Coronaviruses use different domains within the spike S1 subunit and recognize diverse entry receptors (Hulswit et al., 2019; Tortorici and Veesler, 2019). MERS-CoV-S recognizes non-acetylated sialoside receptors (Qian et al., 2013; Park et al., 2019) and binds the dipeptidyl-peptidase four domain (DPP4) receptor (Zhou et al., 2014), while SARS-CoV and SARS-CoV-2 interact directly with angiotensin-converting enzyme 2 (ACE2) through their RBDs (Li, 2013; Shang et al., 2020; Zhang H. et al., 2020).

In SARS-CoV S1 a small fragment of 18 amino acids located into RBD (473-N … … 491-Y) (Ortega et al., 2020) is responsible of this recognition and eight of these 18 amino acids are also preserved in SARS-CoV-2^49^. SARS-CoV-2 RBD adopts two distinct conformations that are defined “up” to represent the receptor accessible state and “down” conformation for inaccessible state. The structures of RBD-ACE2 complexes show that the RBD folds around a five-stranded β sheet and interacts with the N-terminal lobe of ACE2, mainly by hydrophilic interactions (Cai et al., 2020). Very recently, cryogenic electron microscopy (cryo-EM**)** structures of the full-length S protein, showed its pre-fusion and post-fusion conformational states. In detail, in absence of ACE2, when the trimeric protein S is cleaved by furin proteases, it dissociates and adopts a post fusion conformation. The same occurs upon different detergent treatments (NP-40 or n-dodecyl β-D-maltoside (DDM)), suggesting that the S protein dissociation is triggered mainly by hydrophobic interactions and that the ability of S trimer to bind ACE2 is higher than monomers (Cai et al., 2020). In truth, cryo-EM analysis has been performed also for other coronaviruses (e.g., MERS, HCoV-229E) and structures for both conformations (pre and post-fusion) have been provided (Kirchdoerfer et al., 2016; Walls et al., 2016b; Gui et al., 2017; Walls et al., 2017; Kirchdoerfer et al., 2018; Shang et al., 2018; Li et al., 2019).

A different mechanism was instead studied for SARS-COV where the binding with ACE2 is essential to promote the release of S1 subunits from the S trimer and to allow the protein post fusion conformational transition (Song et al., 2018).

Significantly, higher antibody binding titers against S2 have been discovered in COVID-19 patients with respect to those against the entire RBD and S1, suggesting that S2 is more exposed than these regions (Wu et al., 2020).

In S2 region, the furin-like S2’ cleavage site (KR↓ serine phenylalanine (SF) where P1 and P2 are basic residues and P2′ is a hydrophobic phenylalanine) has been reported to be equal between SARS -CoV and SARS-CoV-2 (Table 1), while it is substituted by the sequence RXXR↓S alanine (A), with P1 and P4 as basic residues and an aliphatic alanine residue at P2′ position in MERS-CoV and HCoV-OC43 (Table 1) (Coutard et al., 2020), (Figure 1C).

Unlike S2′ site, the S1/S2 one shows several variances in its sequence among different coronaviruses as shown in Table 1. Moreover, for SARS-CoV-2, it has been suggested the presence of a novel furin-cleavage S1 site (Figure 1B) which contains a solvent-exposed PRRAR↓S valine(V) fragment (Braun and Sauter, 2019; Izaguirre, 2019) able to provide an efficient spreading in the human population as compared to the other betacoronaviruses (Millet and Whittaker, 2014). Structural proteins play also important roles in the replication cycle (Siu et al., 2008; Schoeman and Fielding, 2019); indeed, N protein, localized near the Golgi complex, can be transiently expressed to enhance the production of virus-like particles (VLPs). Moreover, it is also involved in assembly, growth and complete virion formation (Tooze et al., 1984; Klumperman et al., 1994; Siu et al., 2008). Human beta CoV N proteins’ alignment shows that viruses have only 99 similar positons and 83 identical one,with a similarity of 17.256% that increases up to 40.492% for SARS-CoV/CoV-2 and MERS viruses (Coronaviridae Study Group ICTV, 2020).

The M protein is the most abundant structural protein that defines the shape of the viral envelope and it interacts with other structural proteins (Neuman et al., 2011). Its homotypic interactions allow virion envelope formation but, to bring it to completeness, interactions with both S protein, for its incorporation into new virions, and N protein to stabilize the nucleocapsid or the virion internal core are also required (Escors et al., 2001; Neuman et al., 2011; Fehr and Perlman, 2015). Furthermore, through the E protein, the viral envelope is completed and the VLPs are produced and then released (Corse and Machamer, 2003). It has been suggested that mutations in the N-terminus region of M protein, exposed on the virus surface, could play a key role in the host-cell interaction (Maginnis, 2018). H beta CoVs shows a great variability in M proteins with a similarity of only 26.087% and around 60% of overlapped regions. As to N protein, in SARS-CoV, CoV-2 and MERS alignment the similarity increases up to around 40% (Coronaviridae Study Group ICTV, 2020; Gussow et al., 2020).

The E protein is the smallest structural protein localized at the site of intracellular trafficking such as the endoplasmic reticulum (ER), Golgi and the ER-Golgi intermediate compartment (ERGIC), where it contributes to CoV assembly and budding (Venkatagopalan et al., 2015). Recombinant CoVs lacking of E protein exhibit reduced viral titers and blocked viral maturation, corroborating its fundamental role in virus production and maturation (Schoeman and Fielding, 2019). Recent studies outlined that E protein is highly similar to those of bat and pangolin coronavirus, even if in humans it seems to possess unique modifications and characteristics. In particular, an Arg residue is replaced by Glu or Gln at the C-terminal side and a unique deletion flanks this residue. Unfortunately, it is not yet known whether these mutated sites are exposed to the internal or external side of the membrane. This mutation has been suggested to have significant implications for conformational properties and for protein-protein interactions, but additional structural studies are needed (Bianchi et al., 2020). Alignments between E proteins derived from human beta CoVs show a great variability with a similarity of only 9% that reaches the 34% in SARS related viruses (Gussow et al., 2020).

## COVID-19: Actual Strategies for Vaccine Development

Multiple efforts are in progress to achieve safe and efficient vaccines and hundred research laboratories are experimenting, simultaneously, different vaccination strategies (Callaway, 2020; Le et al., 2020; Le et al., 2020). By late-June 2020, over 170 vaccine candidates are in development, with 33 in clinical evaluation: six in phase III, three in phase II (efficacy and dose-testing studies in human subjects), 14 in phase I–II (safety and efficacy trials) and 10 in phase I (Mullard, 2020; World Health Organization, 2020; Robbiani et al., 2020).

Different ongoing strategies are schematically described as follows:

**A. *Virus-based vaccines*** include weakened or inactivated viruses.

- **Weakened virus.** Attenuated vaccines contain laboratory-weakened forms of the pathogenic agent and generate strong cellular and humoral responses (Chen et al., 2020). These type of vaccines typically produce long-term immunity with few doses. The main disadvantage is that live microorganisms are potentially risky: a reversion to the original virulence is sometimes possible (Clem, 2011).
- **Inactivated virus.** The inactivation of the pathogens is obtained through chemical or physical methods rendering the vaccine stable but, often, stimulated immune response is weak implying several doses of administration (Clem, 2011; Xia et al., 2020). Different inactivated virus vaccines are now in clinical trial for COVID-19 as PiCoVacc, by the company Sinovac, which showed SARS-CoV-2–specific neutralizing antibodies response in different preclinical models (mice, rats, and nonhuman primates) (Gao et al., 2020) and good safety and immunogenicity profile in phase I/II clinical trial; actually it is in phase III.

**B. *Nucleic-acid vaccines*** could involve both DNAs and RNAs, they penetrate the host cells translating for viral proteins that will be processed and presented to immune cells by Antigen Presenting Cells (APCs).

- **DNA vaccines.** They are plasmids (circular DNA) containing a gene encoding for an antigen and a promoter/terminator to allow gene expression in mammalian cells. The major advantage is the easiness of manipulation to induce efficient B- and T-cell responses but often they present lower immunogenic responses in humans with respect to other vaccines, as those based on proteins, that, in turn, could be integrated into host’s DNA (Liu, 2003). INO-4800 is a synthetic DNA-based vaccine encoding for SARS-CoV-2 S protein, able to provide efficient immunization in preclinical models (Smith et al., 2020); actually in phase I/II clinical trial.
- **RNA vaccine.** Two types of RNA are currently used: non-replicating mRNAs, whose main advantage consists in direct injection; self-amplifying RNA (SAM), where the genes encoding for structural proteins are replaced with those encoding for the antigens of interest. SAM vaccines are able to create their own adjuvants in the form of dsRNA structures, but present many side effects (Pardi et al., 2018). Two mRNAs lipid encapsulated into nanoparticle (LNP) are currently in phase III trial: mRNA-1273 (Wang F. et al., 2020) and BNT162b2 (Walsh et al., 2020) vaccines.

**C. *Viral-vector vaccines*** employ unrelated and modified viruses encoding for one or more antigens. This technology either utilize **live** (replicating but often attenuated) or **non-replicating vectors**. Adenovirus, measles virus and Vesicular Stomatitis Virus (VSV) are among the most employed viral vectors (Rauch et al., 2018). Several COVID-19 vaccines in phase III, use adenoviral vectors, expressing the S glycoprotein as ChAdOx1 that provided significant results in terms of T-cell response and neutralizing antibodies production (Folegatti et al., 2020); Ad5 vectored COVID-19 vaccine, that demonstrated high tolerability and immunogenicity as reported in trial phase I studies (Zhu F.-C. et al., 2020).

**D. *Protein-based vaccines*** include different types: protein subunits, Virus-like particles (VLP) and peptides.

- **Subunit vaccines** are constituted by pathogenic proteins or polysaccharides purified from natural sources or expressed by recombinant DNA methods. They utilize only specific antigens of the virus, avoiding the onset of adverse reactions but an important issue is to determine the most immunogenic antigenic subunits (Clem, 2011). The company Novavax proposes NVX-CoV2373 as subunit vaccine, that now is in phase II and is composed by S protein and Matrix-M1 adjuvant (Keech et al., 2020).
- **Virus-like particles** are composed by envelope and/or capsid proteins from many viruses, without genetic material. VLPs' advantage consists in similar structures and antigenicity of the pathogen without danger even if they are difficult to manufacture (Grgacic and Anderson, 2006). Only one VLP vaccine, developed by Medicago Inc., reached the phase I clinical trial. It is a plant-derived VLP adjuvanted vaccine that employs living plants as bioreactors to produce non-infectious versions of viruses. (https://www.medicago.com/en/covid-19-programs/).

Herein we focus on peptide-based vaccines describing their advantages or disadvantages with a major emphasis on their current status to fight COVID-19.

## Peptide-Based Vaccines in Diseases

**FIGURE 2:**
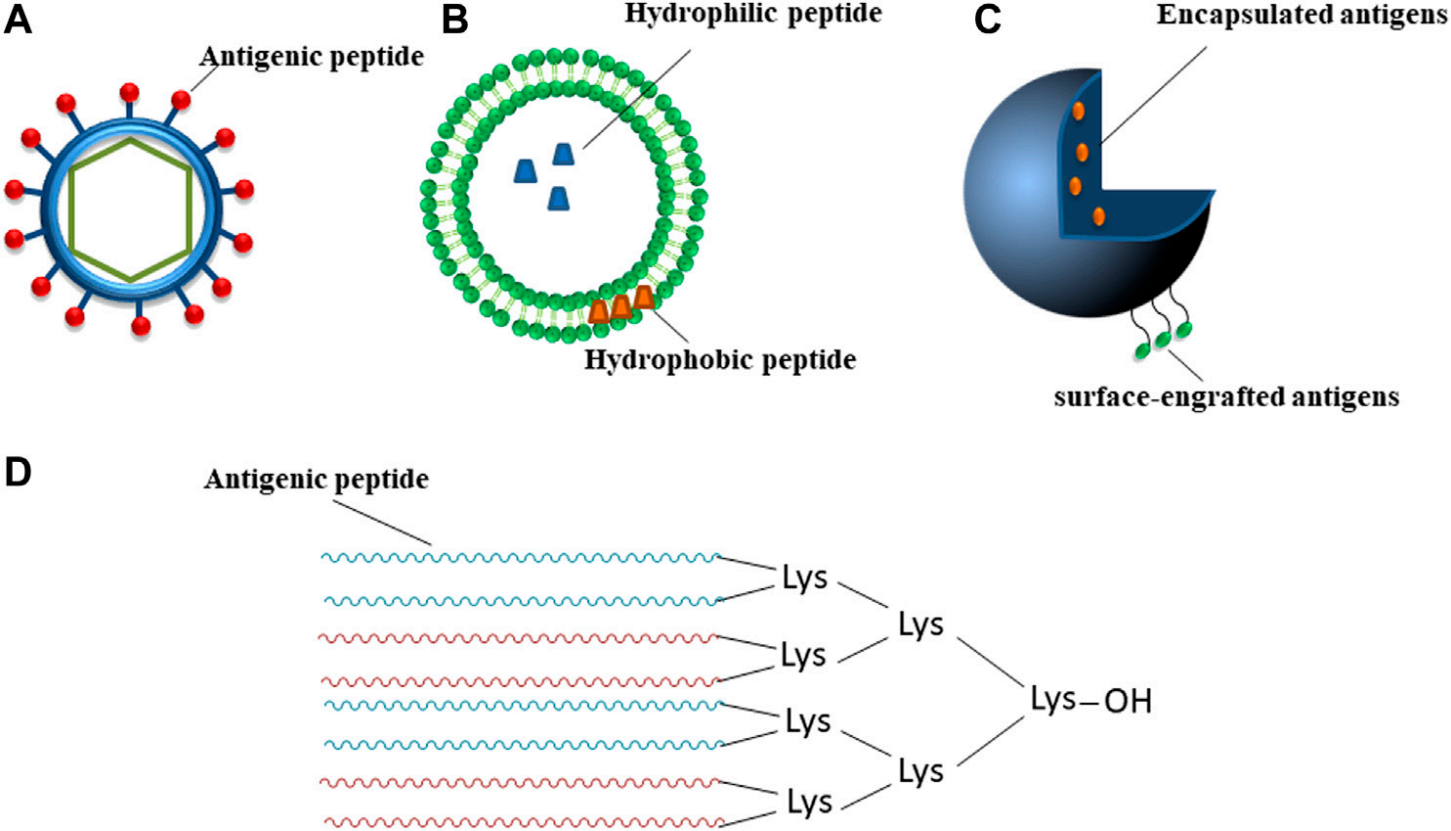
Schematic representation of main types of vaccine delivery systems. **(A)** VLP, **(B)** liposome-based particle, **(C)** polymer micro-/nano-particle, **(D)** MAP, different colors indicate peptide sequences that can be diverse; “OH” is a free/unblocked carboxyl group.

An innovative approach is represented by peptide-based prototypes (Skwarczynski and Toth, 2016; Malonis et al., 2020) that are often able to overcome disadvantages encountered by other strategies as a more safe profile since for easiness of purification (Marasco et al., 2008; Marasco and Scognamiglio, 2015). Their chemical synthesis renders them suitable for large scale production with low costs and high reproducibility (Tizzano et al., 2005). Normally, they are also soluble in water and more stable in storage conditions (Li et al., 2014) but they can be unstable in the body and easily degraded by proteases before eliciting an efficient immune response. Furthermore, they are usually weak immunogens and need adjuvants (additional immune stimulants) to stimulate B- and T-lymphocytes and to induce an effective response. Peptide-based vaccines are usually made of synthetic B- or T- cells epitopes (class I or class II) that can also be combined. T-cells recognize peptide sequences complexed with major histocompatibility complex (MHC) class I or II molecules on the surface of APCs. CD8^+^ T-cells (also called cytotoxic T lymphocytes (CTL)), kill infected cells, while CD4^+^ T helper cells, that recognize MHC II-epitope complex, interact with CTL to reinforce their activity and with B-cells to activate the production of specific antibodies against the pathogen (Sanchez-Trincado et al., 2017). Epitope mapping represents the identification of the binding site, i.e. “epitope”, of an antibody on its target antigen (Gershoni et al., 2007). The most practical strategy relies on the employment of *in silico* computational methods (Hager-Braun and Tomer, 2005) (as schematically reported in Figure 2, applied to SARS-CoV-2); once identified, peptide epitopes are usually modified in order to optimize their functional properties, to enhance immune recognition and to elicit significant immune responses. Ala-scan or combinatorial approaches could be performed to investigate the role of each amino acid residue in antigen/antibody recognition and hence linear flank-modified or cyclic epitopes can be obtained also through stapling insertions (Lau et al., 2015; Godel et al., 2012). Such epitopes, once improved in their antigenic properties, can be included in specific delivery carriers to protect them from degradation (Allahyari and Mohit, 2016): they include VLPs, liposomes, polymeric micro-/nano-particles and dendrimeric systems that are schematically described in Figure 2 (Neek et al., 2019; Singh et al., 2007). In VLPs, (Figure 2A), the antigen can be immobilized on the surface through biochemical tools or covalent links (Sapsford et al., 2013; Qian et al., 2020; Koho et al., 2015; Syomin and Ilyin, 2019). The advantages of VLPs include similar size and antigenicity of the corresponding viruses and lack of viral genome and, as consequence, of danger. A VLP modification is constituted by VLPs assembled from the coat protein of bacteriophages (Bao et al., 2019; Manoutcharian, 2005; Hess and Jewell, 2020). In liposomes, antigen can be entrapped inside them (soluble antigens) or incorporated into lipophilic bilayer (lipophilic antigens) (Figure 2B) (De Serrano and Burkhart, 2017). Also, polymeric micro and nanoparticles can contain encapsulated and/or surface-engrafted antigens with shape and size similar to the virus (Figure 2C). On the basis of the percentage of biopolymers, among others chitosan and poly(lactic-co-glycolic acid) (PLGA) (Duran et al., 2019), it is possible to obtain controlled release from micro/nanoparticles able to extend antigen interaction with the immune cells to induce an effective immune response (Sadler and Tam, 2002). Dendrimers, as polyamidoamine (PAMAM), polypropyleneimine (PPI) and multiple antigenic peptides (MAPs) (Figure 2D) are hyper-branched polymers used for the delivery of different therapeutics (Sadler and Tam, 2002). Due to their well-defined, multifunctional and stable construct, the use of dendrimers has a dual purpose: 1) multi-copies of antigenic peptides usually enhance immunogenic response and 2) their unnatural structure is stable to proteases degradation and can be used for delivery (Joshi et al., 2013). They are conceived in two forms: 1) homotropic that contains multimers of the same peptide or 2) heterotropic, obtained by the combination of different epitopes, used for example for vaccine development of classical swine fever *virus* (CSFV), a typical B_4_T-type vaccine containing one T-cell epitope from the NS2-3 protein linked to four copies of B-cell epitopes from the E2 protein (Monso et al., 2011; Joshi et al., 2013).

### Peptide-Based Vaccines in Clinical Trials for Wide-Impact Diseases

Peptide-based vaccines have been under investigation for several decades. The earliest peptide vaccination study was reported by *Anderer and co-workers* in 1963. They observed that a peptide of six amino acids corresponding to the linear sequence of tobacco mosaic virus could elicit virus-neutralizing antibody. However, only in 1980 the immune response to peptides has been investigated in detail (Arnon, 1972; Brown, 1994). Several researchers observed that degradation of proteins into small peptides was crucial for their presentation to T-cells and was necessary for the antigen-processing events. Moreover, it was observed that peptides could stimulate protective immune responses against some viruses, such as hepatitis B (Li et al., 2014). These results prompted researchers to study if it would be possible to apply this knowledge to make vaccines. Since 1990, over 100 synthetic peptide vaccines have entered phase I and II clinical trials but none of them is currently available on the market. These failures are due to several problems that characterized these vaccines, such as insufficient immunogenicity, chemical instability due to degradation and conformational instability (Chen et al., 2009). The continuous research has however allowed to overcome many of these obstacles. For example, the problem of low immunogenicity is now overcome thanks to several multiple antigenic peptides, moreover, different innovative administration routes (e.g., intradermal, oral, intranasal) (Wang et al., 2015; Waghule et al., 2019) and formulations (e.g., liposomes, nanoparticles) are now able to impair peptide degradation (Singh et al., 2020). Many efforts have yet to be made to obtain a valid peptide-based vaccine, but currently different peptide-based vaccines are under investigation in clinical trials for several diseases as reported in Table 2 and seven of them are actually in phase III clinical trials.

**TABLE 2:** Worldwide clinical trials of peptide-based vaccines (source http://www.clinicaltrials.gov, website access 20/07/2020).

Trial phase	Number of peptide-based vaccines	Clinical indications
I	178	Cancer, HIV infections, autoimmune diseases, arthritis, digestive system diseases, gonadal disorders, lung diseases, RNA virus infections, skin diseases, malaria, allergy, mycoses, influenza, hepatitis, hand, foot and mouth disease
II	115	Cancer, blood coagulation disorders, liver diseases, lung diseases, bone marrow diseases, endocrine system diseases, hepatitis, skin diseases
III	7	Cancer
IV	0	No peptide vaccine reached market yet

As examples of peptide-based vaccines that have reached the phase III, we outline here scientific contexts and design procedures to achieve promising vaccines.

NeuroVax^™^ is a therapeutic TCR (T-cell receptor) peptide vaccine for secondary progressive multiple sclerosis (SPMS). T-cells specific for myelin antigens, particularly myelin basic proteins (MBPs), play a crucial role in the immune response in MS: high MBP levels were detected in blood and cerebrospinal fluid (CSF) of patients (Lamers et al., 1995). The actual therapeutic strategy is to enhance regulatory power of T-cells: three TCR families, mainly β variable (BV) five and BV6 and to a lesser extent BV13S1, are normally expressed by MBP specific T-cells in the blood, CSF and brain of MS patients (Kotzin et al., 1991). In order to develop therapeutic TCR peptides, two CDR2 (complementarity-determining regions 2) protein region of TCRVβ (T-cell receptor β variable) 5.2 and 6.1, BV5S2 (39–59) (ALGQGPQFIFQYYEEEERQRG) and BV6S1 (39–59) (LGQGPEFLIYFQGTGAADDSG), corresponding to TCR sequences over-expressed by MBP-specific T-cells, were tested (Chou et al., 1994). Both peptides demonstrated able to reduce MBP-specific T-cells by enhancing TCR-reactive T-cells. To find residues of BV5S2 crucial for the binding affinity toward human leukocyte antigens (HLA)-DR2 alleles that are associated to MS, overlapping peptides of BV5S2 (1–94) were also examined. The fragment 38–58 of BV5S2 with the mutation Thr (Ortega et al., 2020) was the most immunogenic, suggesting that position 49 is a TCR contact residue. Another study revealed that also the TCR BV6S5 chain and, in particular the peptide corresponding to BV6S5 39–58 fragment (LGQGPEFLTYFQNEAQLEKS), possesses immunogenic properties in almost 90% of MS patients used in the study. For the formulation, this peptide was emulsified in incomplete Freund’s adjuvant (IFA), an adjuvant used to make a water-in-oil emulsion (Gold et al., 1997). Based on these results, NeuroVax, a trivalent TCR peptide formulation in IFA of [Thr49] BV5S2(38–58), BV6S5(39–58), and BV13S1(42–60) CDR2 peptides was developed (Bourdette et al., 2005). Interestingly, this vaccine was able to increase the numbers of circulating IL-10-secreting T-cells, reactive to TCR peptides, in MS patients (Vandenbark et al., 2008; Arevalo-Villalobos et al., 2020).

Proteinase 1 (PR1) is a peptide vaccine in phase III for leukemia. Primary granule proteins (PGPs) are serine proteases found principally in granulocytes (Wiedow and Meyer-Hoffert, 2005). Two PGPs, PR3 and neutrophil elastase (NE), are overexpressed in myeloid leukemia blasts and CD34^+^ leukemic progenitors. Moreover, it was observed that both PGPs can act as leukemia antigens, inducing a protective immune response; indeed, in patients with high levels of both PGPs, better outcomes after allogeneic stem cell transplant were observed (Weber et al., 2013). On these bases and using reverse immunology (prediction of immunogens starting from the sequence of the gene of interest), a nonapeptide named PR1 (VLQELNVTV) was identified. This is a HLA-A2-restricted peptide shared by both PR3 and NE enzymes (Rezvani, 2008). In I and II clinical phases, PR1 vaccine revealed no toxicity and no adverse autoimmune reactions; additionally, decrease of disease activity was found in 33% of patients (Qazilbash et al., 2017). Actually, this vaccine is in phase III clinical trials in association with sargramostim, a recombinant granulocyte macrophage colony-stimulating factor (GM-CSF), normally used to cure neutropenia caused by chemotherapy during the AML treatment (source http://www.clinicaltrials.gov).

## Peptide-Based Vaccines in COVID-19

### Design of Peptide-Based Vaccines Against Syndrome Coronavirus 2

Cytotoxic T-Lymphocyte (CTL) vaccination was proposed by employing multiple class I epitopes in different infections (Woolard and Kumaraguru, 2010). In a recent study this approach was applied following a parallel way between SARS-CoV-2 and Ebolavirus (EBOV), a nine amino acid peptide, corresponding to the fragment NP44–52 of EBOV N protein, revealed immunogenic *in vivo*. PLGA microspheres decorated with the peptide, along with adjuvants, were employed to vaccinate mice models, obtaining high enzyme-linked immunospot (ELISPOT) response (Herst et al., 2020). In survivors to EBOLA infection, the most commonly targeted (by CD8^+^ T-cells) EBOV NP epitope was NP43–53 (11 amino acids), whose protection was previously demonstrated in C57BL/6 mice (Wilson and Hart, 2001). However, the efficacy of NP 43–53 *in vivo* was not so high as expected, thus it was speculated that a smaller region within it, 43–53 fragment, could be more immunogenic and this hypothesis was confirmed by 44–52 peptide (YQVNNLEEI) that proved to be the most immunogenic among few other small peptides. Prompted from EBOV experimental data, it can be assumed that CTL response could be beneficial also in the context of a coronavirus infection (Channappanavar et al., 2014; Liu et al., 2017). A similar investigation was translated on SARS-CoV-2 nucleocapsid (N) protein *in silico*. Actually this protein is considered endowed with strong immunogenicity and multiple functions in regulating viral RNA synthesis during replication and transcription indeed several studies outlined high levels of IgG antibodies against N protein detected in sera from SARS patients (Dutta et al., 2020; Jiang et al., 2020). The screening provided several peptides present on both SARS-CoV and SARS-CoV-2 N phosphoproteins as epitopes that were tested for potentially *in vitro* efficacy as well as HLA restriction (Herst et al., 2020).

Prompted by the lack of time and the urgent need of vaccination in COVID-19 infection, immunoinformatic approaches demonstrated elected tools in the identification of potential epitopes (Garg et al., 2020).

**FIGURE 3:**
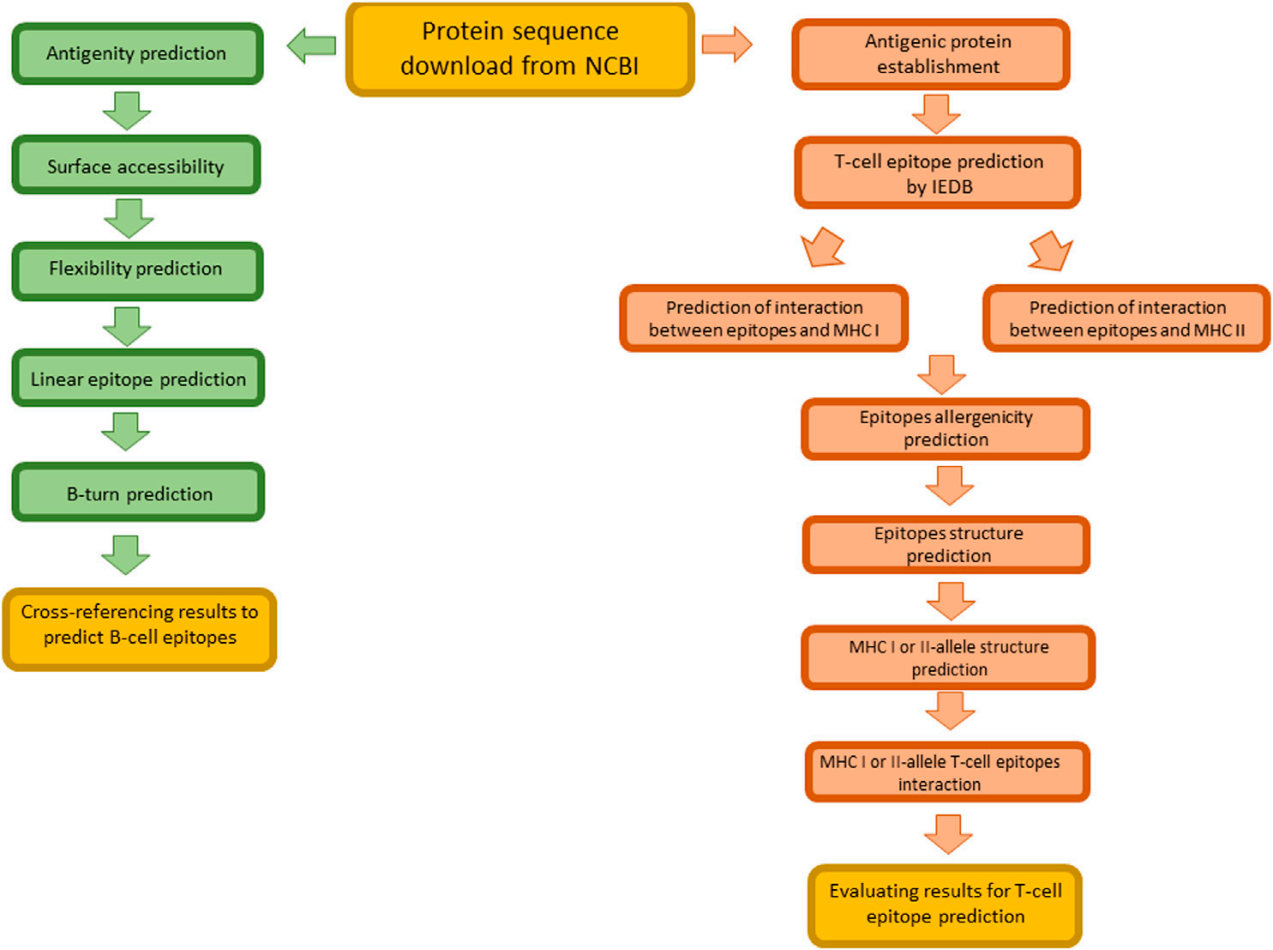
Schematic representation of main steps for the identification of B- and T- cell epitopes *in silico*.

In Figure 3 schematic representation of the main steps for immunoinformatic approach followed in the prediction of epitopes targeting B- and T-cells is reported.

A systematic study reported on the identification of MHC class-I binding-epitopes screening, based on the analysis of 12 MHC‐I super types (groups of MHC molecules) and, at the same time, on TAP (transporter associated with antigen processing protein complex) transport efficiency prediction. In this study, moreover, 162 MHC-II peptides (IC50 ≤ 100 nM) were predicted immunogenic by using binding prediction tools. A screening *in silico* provided three CD8^+^ T-cell peptides and five CD4^+^ T-cell peptides as the most promising for the design of a vaccine (Khan et al., 2020).

Similarly, a multitarget approach was employed to map immunogenic epitopes (B-cell and T-cell) over the entire structural proteins of SARS-CoV-2 and by applying various computational and immune informatic approaches. Hence a multi-epitope peptide-based vaccine was designed and predicted to be highly immunogenic in the largest proportion of world’s human population (Singh et al., 2007).

On the other hand, the design of a COVID-19 vaccine including few epitopes targets was attempted. Among 174 SARS-CoV-2 epitopes with high predicted binding scores, only four peptides not overlapping with T cell-confirmed epitopes from SARS (deposited at IEDB https://www.iedb.org/epitope/). These peptides were validated to bind stably to 11 HLA allotypes (Prachar et al., 2020) class I, II and further tested employing the E protein of SARS-CoV-2 as immunogenic target. They appeared promising candidates for vaccine design with adequate world population coverages (88%) (Abdelmageed et al., 2020).

Besides *in silico* screening of potential epitopes, a great advantage for the design of vaccines could derive from *in vivo* proteomic investigations in COVID-19 patients. In a first study T-cell target antigens were highlighted, in peripheral blood mononuclear cells (PBMCs), and important information on patients' T- cell response were extrapolated, using different experimental approaches (Grifoni et al., 2020). An initial computational approach was employed to predict SARS-CoV-2 T-cell epitopes and then megapools (MPs) of HLA class I and II predicted peptides were generated to test large numbers of epitopes. Libraries of overlapping synthetic peptides, spanning the entire sequences of all proteins of the viral genome (except S protein) were pooled and then separated then their validation in patients’ sera was carried out through ELISA and flow cytometry. Subsequently, SARS-CoV-2 open reading frame (ORF) proteins targeted by CD4^+^ and CD8^+^ T-cells were examined. T cell receptor (TCR) dependent activation induced marker (AIM) assays was employed to identify and quantify SARS-CoV-2-specific CD4^+^ T-cells in COVID-19 patients, while for CD8^+^ T-cells both AIM and intracellular cytokine staining (ICS) assays were used. ICS was useful to detected IFN-γ^+^ SARS-CoV-2- specific CD8^+^ T-cells. In the examined cohort of patients both CD4^+^ and CD8^+^ T responses were observed, even if the most frequent were CD8^+^ T (Grifoni et al., 2020).

Another study reported on the identification of two immunodominant linear B-cell epitopes, belonging to the S glycoprotein of SARS-CoV-2. To this purpose, the antibody profiles of SARS-CoV patients and COVID-19 convalescent patients were analyzed (Poh et al., 2020). The analysis of a linear B-cell peptide library spanning the entire S of either SARS-CoV-2 or SARS-CoV, in pools of five overlapping peptides allowed to identify several epitopes. Among these, two different peptide pools from SARS-CoV-2 library, named S14 and S21, were strongly detected by COVID-19 patients' sera but not by SARS-CoV controls. Further investigations on individual peptides within pools S14 and S21 restricted the analysis to two specific regions of interest: the peptides named S14P5 and S21P2 where the first is a sequence in close proximity to the RBD and the second covers part of the fusion peptide. Conversely, pseudo-typed lentiviruses expressing SARS-CoV-2 S glycoprotein were used to detect neutralizing antibodies in patients’ sera. To test the neutralizing capacity of antibodies directed against S14P5 and S21P2, antibody depletion assay was performed and analyzed by ELISA. Depleted sera for antibodies targeting either peptides S14P5, S21P2, or S14P5+S21P2 peptides led to a significantly reduced ability to neutralize SARS-CoV-2 pseudo-virus infection, when compared to the non-depleted sera control. These results demonstrated that antibodies targeting identified two linear epitopes are significantly involved in the anti-S-neutralizing response (Poh et al., 2020).

Similarly, the *in vivo* antibody-response to RBD-epitopes and S proteins were investigated on blood samples derived from 149 Covid-19 convalescent individuals. Plasmas were collected after 39 days from the onset of the disease and tested for binding to the SARS-CoV-2 RBD and trimeric S proteins by ELISA. To determine the nature of the antibodies elicited by SARS-CoV-2 infection, flow cytometry was employed to isolate individual B lymphocytes with receptors that bound to RBD from the blood of six selected individuals. Then several monoclonal antibodies were expressed in cells and their neutralizing activity was assessed by ELISA and bilayer interferometry experiments were performed. Results show that there are multiple distinct neutralizing epitopes on the RBD of SARS-CoV-2 recognized and neutralized by pseudo-viruses. Even though sera derived from the analyzed cohort of patients did not contain high levels of neutralizing antibodies, rare but recurring RBD-specific antibodies with potent antiviral activity were found in all individuals tested, suggesting that a vaccine designed to elicit such antibodies could be surely effective (Robbiani et al., 2020).

### Peptide-Based Syndrome Coronavirus 2 Vaccine Pharmaceutical Market

Recently World Health Organization (WHO) reported on peptide-based vaccines against SARS-CoV-2 (Table 3) (https://www.who.int/publications/m/item/draft-landscape-of-covid-19-candidate-vaccines). One of the most promising is developed at Valo Therapeutics Ltd., company, applicable to other coronaviruses (https://www.genengnews.com/covid-19-candidates/covid-19-too-soon-to-tell/valo-therapeutics/). This pan-coronavirus vaccine was obtained employing the Peptide-coated Conditionally Replicating Adenovirus (PeptiCRAd) technology and engineered to express coronavirus spike proteins together with HLA-matched peptides to enhance the CD8^+^ T-cell immune responses.

**TABLE 3:** Peptide vaccines in preclinical phase. Source: World Health Organization (WHO) website.

Vaccine	Producer/s	Type of candidate vaccine
FlowVax COVID-19	Flow pharma	Adjuvanted, microsphere peptide vaccine targeting SARS-CoV-2 N with a suite of 16 T-cell peptides
DPX-COVID-19	IMV inc	Peptide antigens formulated in lipid nanoparticles (LNP)
	Vaxil bio	Peptide
	VIDO-InterVac, university of Saskatchewan	Adjuvanted microsphere peptide
	OncoGen	Synthetic long peptide vaccine candidate for S and M proteins
Ii-key peptide vaccine	Generex/EpiVax	T-cell epitopes + ii-key peptides
	University of sao paulo	Vlp peptides
	Axon neuroscience	Peptides derived from spike protein
	Intravacc/Epivax	Outer membrane vesicle (OMV)-subunit
	VIDO-InterVac, university of Saskatchewan	Adjuvanted microsphere peptide
	Valo therapeutics ltd	Adenovirus-based + HLA-matched peptides
	FBRI SRC VB VECTOR, rospotrebnadzor, koltsovo	Peptide vaccine

WHO, World Health Organization; LNP, lipid nanoparticles; OMV, outer membrane vesicle.

PeptiCRAd is an innovative technique used to combine two clinically proven cancer immunotherapy approaches: an oncolytic adenovirus and a peptide vaccine taking advantages of the both approaches (Tähtinen et al., 2020). It was able to adsorb tumor-specific major histocompatibility complex class I (MHC-I) peptides onto the viral surface to drive the immune response toward the tumor epitopes, obtaining a more powerful anti-tumoral immune response (Ylösmäki and Cerullo, 2020). For SARS-CoV-2 vaccine spike proteins should be processed to induce both T-cell and antibody-mediated immunity to COVID-19, since coating the adenovirus with peptides specifically selected for CD8^+^ T-cells increase the cell-mediated immune response broadening the immune targets. Currently, this vaccine is in preclinical phase.

Another example of peptide-based vaccine is under investigation by IMV Inc. company. It is based on the DPX platform, able to activate both B- and T-cells responses (Ye et al., 2020). The DPX is a fully synthetic, lipid-based, delivery platform patented by the company, with no aqueous component in the final formulation. The DPX platform can be formulated with peptide antigens and thanks to its unique “no release” mechanism of action, APCs are attracted to the injection site, aiding a more robust and sustained immune response within lymph nodes (Dorigo et al., 2020). The final product is stored in dry form and reconstituted in lipids for injection, resulting in an extended shelf life and simple handling or administration for clinicians. DPX-COVID-19 could be rapidly produced in large-scale and IMV company stated that the first patient should receive DPX-COVID-19 during the summer of 2020.

The Vaxil corporation declared a vaccine based on unique and patented signal peptide (SP) identified through a bioinformatics platform (Wu, 2020). This strategy provides the possibility to use signal peptide domains on crucial proteins to develop targeted therapies against cancer or infectious disease pathogens. The screened SPs are able to induce a robust T- and B-cell response across wide and varied HLA subtypes acting as universal neoantigens (Kahandal, 2020). Vaxil applied for a US patent (U.S 62/987310) to obtain a broad patent protection for novel vaccines, pharmaceutical compositions and methods to produce a peptide vaccine against coronaviruses (https://www.globenewswire.com/fr/news-release/2020/03/27/2007667/0/en/VAXIL-COMMENCES-PRECLINICAL-COVID-19-VACCINE-TRIAL-AND-FILES-AN-ADDITIONAL-COVID-19-PATENT.html). Their studies are in the preclinical phase.

Flow Pharma generated a compound able to induce a robust immune response by targeting parts of the virus least likely to mutate. FlowVax COVID-19 is a room temperature stable dry powder developed for delivery by injection or inhalation. Despite traditional antibody vaccines that produce an antibody response, recognizing only targets on the surface of the virus, this vaccine is able to recognize many viral targets within an infected host. This kind of T-cell vaccines provide long-lasting immunity, preventing recurrence and can be used for pre-exposure or post-exposure prophylaxis. In the FlowVax COVID-19 formulation, the antigenic peptide is microencapsulated into biodegradable microspheres stable at room temperature. This strategy has already reached good results in Ebola vaccination as previously described (Herst et al., 2020). A similar strategy based on polymeric microencapsulation is being developed by VIDO-Inter Vac company and the University of Saskatchewan but unfortunately no details are available in literature yet.

OncoGen researchers designed novel epitope-based peptide vaccines concerning the most frequent HLA alleles found in the Romanian COVID-19 patients (Tables 3, 4). More in detail, they identified multiepitope peptides able to trigger both CD4^+^ and CD8^+^ T cell immune response. They designed through the Immune Epitope Database (IEDB) server 15/30 mer synthetic long peptides for single S, M or both proteins (Tables 5, 6 **)**, using a cathepsin-sensitive linker (LLSVGG) to bind MHC class I-restricted epitopes to MHC class II-restricted one. The latter is always located at the N-terminal in order to stimulate both CTLs and T helper lymphocytes. The best CD8^+^ and CD4^+^ T-cell epitopes were selected based on their high overall score or affinity for MHC (IC_50_ < 50 nM) (Table 6) (Bojin et al., 2020). This approach is considered by OncoGen as the best compromise between an industrial large-scale global vaccination strategy and an individually-targeted personalized vaccination strategy. A similar strategy is followed by AXON Neuroscience (Novak et al., 2017) consisting into the selection of epitopes able to induce T and B cell-mediated immune responses to prevent interaction of the virus S protein with its target human cells, thus preventing the virus from entering the cells and spreading.

**TABLE 4:** HLA allele frequencies in the Romanian population.

HLA allele	Frequency (%)
HLA-A*02	29
HLA-A*01	14.3
HLA-A*24	11.2
HLA-B*35	16
HLA-B*18	11
HLA-DRB1*11	18.5
HLA-DRB1*03	11.3
HLA-DRB1*13	10.5

**TABLE 5:** CD4^+^ T-cell epitopes, predicted by IEDB.

Protein	Allele	Epitope
S (spike)	DRB1*1101	GNYNYLYRLFRKSN
	DRB1*1301	IRAAEIRASANLAA
	DRB1*0301	INLVRDLPQGFSAL
M (membrane)	DRB1*1101	SYFIASFRLFARTRS
	DRB1*1301	AVILRGHLRIAGHH
	DRB1*0301	EITVATSRTLSYYK

**TABLE 6:** CD8^+^ T-cell epitopes, predicted by IEDB.

Protein	Allele	Epitope	MHC IC_50_ (nM)	Overall score (IEDB)
S (spike)	A*0201	YLQPRTFLL	4.6	1.17
		KIADYNYKL	15.9	0.99
		FQFCNDPFL	8.9	0.99
		SIIAYTMSL	15.3	0.82
		VLNDILSRL	19.7	0.43
	A*0101	LTDEMIAQY	5.2	1.71
		WTAGAAAYY	40.1	0.88
	A*2402	NYNYLYRLF	23.4	1.01
		QYIKWPWYI	8.9	0.45
	B*3501	IPFAMQMAY	2.3	2.24
		LPFNDGVYF	3.5	1.90
		VASQSIIAY	7.2	1.80
		FAMQMAYRF	6.3	1.70
		LGAENSVAY	11.5	1.66
M (membrane)	A*0201	GLMWLSYFI	4.5	0.86
		FVLAAVYRI	11	0.45
		KLLEQWNLV	7.3	0.18
	A*0101	ATSRTLSYY	48.2	0.92
	A*2402	YFLASFRLF	9.9	1.48
		SYFLASFRL	22.5	0.72
	B*3501	YANRNRFLY	6.8	1.66
		VATSRTLSY	23.9	1.27
		FAYANRNRF	23.6	0.99

A combinatorial epitope approach was also used in the development of the **Ii-Key peptide vaccine** *,* and implies the use of a vaccine technology based on hybrid peptides, as proposed by EpiVax, Inc. company in collaboration with Generex Biotechnology Corp (Kallinteris et al., 2006; Wu, 2020). The Ii-Key peptide technology, already adopted for cancer vaccine development, is used to overcome the weakness of presentation of MHC class II epitope vaccine peptides (Kissick et al., 2014). This method assumes that “hybrid molecules” can enhance CD4^+^ T cell response: the hybrid is composed by N-terminus of MHC II epitopes covalently linked, through a simple polymethylene spacer (or other chemical linkers), to the C terminus of the Ii-Key peptide (four amino acids: LRMK). *In vitro,* this chimeric peptide exhibited an enhanced presentation of the antigens of about 200 times in comparison with the epitope alone. *In vivo*, the T- helper cell response is enhanced up to 8 times (measured with ELISPOT test) (Kallinteris et al., 2006); by combining Ii-Key peptide technology with the computational tool for vaccine design, iVAX (De Groot et al., 2020), the resulting vaccine will blend short peptide sequences of the virus with Ii-Key peptides. The aim is to provide broad T cell-mediated protection against SARS-CoV-2 avoiding, at the same time, antibody dependent enhancement (ADE) (Tirado and Yoon, 2003).

In fact, peptide epitopes vaccines can mitigate the risk of ADE, that, instead, was encountered in several viral infections (Smatti et al., 2018). It can occur when the vaccinated patient is exposed to another serotype of the same virus: in this case, the formed antibodies are not only unable to neutralize the virus, but they even enhance virus entry into the host cells. In fact, the antibodies first bind to the virus, and later bind to the IgG Fc receptors on immune cells, mediating viral entry into these cells (Willey et al., 2011; Kuzmina et al., 2018).

It can be speculated that ADE mechanism, exhibited from other coronaviruses (Kam et al., 2007; Jaume et al., 2011; Wang et al., 2014), could occur also for SARS-CoV-2 virus. In another recent study, Mersmab-1, an antibody against RBD of S protein of MERS-CoV efficiently neutralized virus entry by competition with dipeptidil peptidase 4 (DPP4), even if at low concentrations demonstrated to mediate viral entry into the cells (Wan et al., 2020).

Epivax company is also involved in the development of a second SARS-CoV-2 peptide-based vaccine together with Intravacc corporation. Their idea is to combine the safe and immunogenic Intravacc - outer membrane vesicle (OMV) delivery platform with synthetically produced COVID-19 T cell epitopes designed and optimized by EpiVax. OMVs are spherical particles with intrinsic adjuvating properties which can be decorated with immunogenic peptides able to drive effective adaptive immunity (Wu, 2020).

## Conclusions

In this review, we presented an overview of the current state of knowledge on peptide-based vaccines and therapeutics for the SARS-CoV-2 pandemic, summarizing the most recent and possible therapeutic strategies that could have the possibility to pass different clinical trials. We also focused on structural viral proteins being these the most involved when designing SARS-CoV-2 vaccines. Recently, alternative and more selective viral therapies based on peptides have been established (Agarwal and Gabrani, 2020; Brice and Diamond, 2020; Kalita et al., 2020). Peptides are excellent lead compounds for their high specificity even if they present poor plasma stability and oral bioavailability (Conibear et al., 2020). To overcome these limitations new delivery systems together with novel administration routes have been developed making peptides excellent candidates for the pharmaceutical market.

## Author Contributions

Conceptualization, CDN and DM; writing—original draft preparation, DM, CDN, SLM, IDB, and PB; writing—review and editing, DM, CDN, SLM, IDB, and PB; visualization, CDN, SLM, and IDB.

## Funding

This work was partially supported by POR CAMPANIA FESR 2014/2020 “Combattere la resistenza tumorale: piattaforma integrata multidisciplinare per un approccio tecnologico innovativo alle oncoterapie-Campania Oncoterapie” (Project No. B61G18000470007). CDN was supported by the “IBSA Foundation for scientific research,” SLM was supported by AIRC fellowship for Italy.

## Conflict of Interest

The authors declare that the research was conducted in the absence of any commercial or financial relationships that could be construed as a potential conflict of interest.
